# Supplementary material for: Elemental mapping in single-particle reconstructions by reconstructed electron energy-loss analysis
Source: Nat Methods. 2024 Oct 24;21(12):2299–306. doi: 10.1038/s41592-024-02482-5 (PMC11621030; doi:10.1038/s41592-024-02482-5)
Supplement: Supplementary file 1 — Supplementary scripts for acquisition and processing. [file 41592_2024_2482_MOESM1_ESM.pdf]

nature methods

Supplementary information https://doi.org/10.1038/s41592-024-02482-5

Elemental mapping in single-particle reconstructions by reconstructed electron energy-loss analysis

In the format provided by the authors and unedited

Scripts for acquisition and processing

ACQUISITION

SerialEM macro:

```
MacroName SPASTEM

# SPA macro for STEM-EELS spectral images
# Adapted from Wim Hagen's SPA macro
# August 2022
# Olivia Pfeil-Gardiner and Bonnie Murphy

#########################
####### SETTINGS #######
#########################

# Buffer P is expected to have reference hole image from Low Dose View; In this case we want an image centered on the carbon where the focus area should be; which is how we will set up positions in the navigator, too.
# If you are running this script for the first time in your SerialEM version, Run SetCustomTime SI_Timer once in a one line script. The variable is then saved in your properties.

scantime = 12       #in minutes; Time beofre SerialEM continues to next navItem (should be larger than time it takes for SI scan)
totalSItime = 23    #in minutes; Time between start of previous SI scan and start of current SI scan (should be longer than time for acquiring and saving SI in PanthaRhei)
#############################
####### END SETTINGS #######
#############################


SuppressReports
MoveStage -0.025 -0.025
ReportNavItem

SetColumnOrGunValve 1

####### Align to hole #######

CallFunction HoleCentering

####### Focus #######

GoToLowDoseArea F

G #Autofocus

####### Acquire images #######

GoToLowDoseArea R
SetBeamBlank 0

#check if enough time has elapsed since triggering the last SI in PR
ReportCustomInterval SI_timer
If $repVal1 < $totalSItime
extradelay = $totalSItime - $repVal1
```

```
delay $extradelay min
Endif

#CallScript
CreateProcess  C:\path\to\cd_and_acquireSI.cmd
SetCustomTime SI_Timer
delay $scantime min


Echo ===> Done ...
Echo ====================================================
```

Intermediate script: cd_and_acquireSI.cmd

```
cd C:\path\to\RPC_script\
SI_acquisition_client.py >> report.txt
```

RPC script: SI_acquisition_client.py

```
import json
import socket
import os
import time

HOST = 'ip.of.pc'
PORT = portnr

########SET VARIABLES#########
fn = 'File_name prefix__'
filepath = "D:/path/for/saving/"
number_length = 3    #For files named *001.hyspy and so on
extension = '.hspy'
###############################


def call( name, **kwargs):
    with socket.socket(socket.AF_INET, socket.SOCK_STREAM) as s:
        s.connect((HOST, PORT))
        request = json.dumps({
            'jsonrpc': '2.0',
            'id': 42,  # no concurrent calls, the ID does not matter
            'method': name,
            'params': kwargs})
        data = request.encode('utf-8')
        netstring = b'%i:%s,' % (len(data), data)
        print('SENDING:  ', netstring)
        s.sendall(netstring)

        length_string = b''
        while True:
            character = s.recv(1)
            if character == b':':
                break
            length_string += character
        length = int(length_string)
        result = b''
        while len(result) < length:
            received = s.recv(length-len(result))
```

```
            result += received
        print('RECEIVED: ', result)

        if s.recv(1) != b',':
            raise ValueError('Received invalid netstring.')

        reply = json.loads(result.decode('utf-8'))
        if reply['id'] != 42:
            raise RuntimeError('Received unexpected reply')
        if 'error' in reply:
            if 'data' in reply['error']:
                error = ': '.join((
                    reply['error']['message'],
                    reply['error']['data']['message']))
            else:
                error = reply['error']['message']
            raise RuntimeError(error)

        return reply['result']


#################################################################

###### Recreate the name of the previous file to watch before acquiring:###

with open('index.txt') as f:
            fileindex = f.read()

oldname = "D:\\path\\for\\saving\\"+fn + fileindex.zfill(number_length) + extension

#################################################################
###### Increase index and create new filename###

with open('index.txt','w') as f:   #Note: index.txt is a text file that contains only 0 at the beginning of the session
            fileindex = int(fileindex) + 1
            f.write(str(fileindex))

filename = fn + str(fileindex).zfill(number_length) + extension

#################################################################
###### wait until the previous file exists and stops increasing size
print(oldname)
while not os.path.exists(oldname):
            time.sleep(1)

oldsize=-1
while os.path.getsize(oldname)!=oldsize:
            time.sleep(5)
            oldsize=os.path.getsize(oldname)

#################################################################
###### Create a command to send to PR
savecommand = "from panta_rhei.gui.panta_rhei_interface import PantaRheiInterface\nfrom
panta_rhei.scripting import export_to_file\nrepo_name = 'DataCube1'\nfile_path =
"+"'"+filepath+filename+"'"+"\ninterface = PantaRheiInterface.instance()\ndata =
interface.data_from_repo(repo_name)\nexport_to_file(data,file_path)"

#################################################################
###Call the PR command
call('acquireSpectralScan',samples=[4096,4096],imageName='DataCube1')
```

```
call('executeScript',scriptString=savecommand)
```

PROCESSING

Extracting zero-loss micrographs

Running script:

```
#!/bin/bash
inputdir=$1 #Input folder where are the .hspy files (Without / in the end) MAKE SURE TO HAVE THIS PATH
CORRECTLY ASSIGNED AFTER CALLING THE SCRIPT WHEN RUNNING sbatch command
echo $inputdir

cd $inputdir
conda activate hyperspy  # your hyperspy conda environment


echo "Extracting ZLP images from .hspy files, integrating and converting to .tiff"
outputdir=/your/output/dir/ #Change the path here to correspond with your output folder
echo $outputdir

for file in $inputdir/*.hspy
do
	filename="$(echo $file | sed "s#$inputdir/##"| sed "s#.hspy##")" #sed replaces the string btw parentheses to
another (remove the extension and the inputdir)
	echo "File" $file "/Filename" $filename "/Outputdir" $outputdir
	python /path/to/zlp_hspy2tiff.py $file $filename $outputdir #Change the python path here to locate your
file
	echo "File saved as" $new".tiff"
	echo " "

done

echo "done"

########################################
			END
#########
#############################
```

Extraction script:

```
#!/usr/bin/env python

####################
# # 1. Initializing the Hyperspy and libraries
####################

import sys
import hyperspy.api as hs
import numpy as np
import matplotlib.pyplot as plt

####################
# # 2. Loading the Data
####################
```

```
file=sys.argv[1]
filename=sys.argv[2]
master_outputdir=sys.argv[3]

print(' ')
print(">>>"+str(filename)+" processing: Initializing.<<<")
print(' ')
s = hs.load(file)
print('1.Your file was loaded:'+str(s))

######################
### 3. Let's estimate the offset of the ZLP:
######################

s.set_signal_type('EELS')
s_zlp = s.estimate_zero_loss_peak_centre()
s_zlp_mean = s_zlp.mean().data[0]
print('3.ZLP mean offset calculated:'+str(s_zlp_mean)+'eV.')

######################
# # 4. Integrating ROI in the images
######################

s_ti = s.isig[s_zlp_mean-3.5:s_zlp_mean+3.5].integrate1D(axis="Energy")
print('4.ZLP(+-3.5eV) intensities integrated')

######################
# 5. Saving the images as tiff
######################

s_ti2 = s_ti.as_signal2D((0,1))
s_ti2.change_dtype('float32')

########################
#5.1 There is a problem with the metadata of newer data when saving tiff. Delete the problematic information:

s_ti2.axes_manager[0].scale=1
s_ti2.axes_manager[1].scale=1
s_ti2.axes_manager[0].units=''
s_ti2.axes_manager[1].units=''
#########################

e_foldername = str(master_outputdir)
e_filename = str(filename)+'.tiff'
s_ti2.save(e_foldername+e_filename, overwrite=True)
print('5.File saved as: '+e_filename+' in '+e_foldername)

######################
# # 6. Check if this worked:
######################

print('6.Tiff file attributes')
print(hs.load(e_foldername+e_filename).axes_manager)
```

Extracting micrographs for all energies

Running script:

```
#!/bin/bash

###
#Define Variables
###

Inputdir=/path/to/folder/  #Folder where the .hspy files are (With / in the end)
outputdir=/path/to/output/folder/
script=/path/to/unbin_extract_all_energies.py
waittime=30 #time between slurm submissions in s (depends on processing time of one mic and how many jobs
you want to run parallely)

echo $inputdir
cd $inputdir

#
#Create Output Directories
#

mkdir $outputdir
mkdir ${outputdir}Spectra/
echo $outputdir



#
#Loop over Micrographs
#

for file in ${inputdir}*.hspy
do
   filename="$(echo $file | sed "s#$inputdir##" | sed "s#.hspy##")" #sed replaces the string btw parentheses to
another
   echo "File" $file "/Filename" $filename "/Outputdir" $outputdir
   command="$script $file $filename $outputdir"
   echo " "

#
#Edit slurm script, submit, and wait
#

   sed -i  "s#placeholder#$command#" extract.sh
   sbatch extract.sh
   sleep ${waittime}
   sed -i "s#$command#placeholder#" extract.sh

#
#Loop End
#

done
```

Slurm script for cluster submission:

```
#!/bin/bash

#SBATCH --error=E-Extract.err
#SBATCH --output=E-Extract.out
#SBATCH --mincpus=1
#SBATCH --cpus-per-task=1
```

```
#SBATCH --ntasks=1
#SBATCH --qos=normal
#SBATCH -J »--->Energy_Extract<---«
#SBATCH --open-mode=append

# Print the start time
echo "SLURM JOB SUBMITTED " `date '+%Y-%m-%d %H:%M:%S'`

conda activate hyperspy

srun -n 1 `which python` placeholder

seff $SLURM_JOB_ID

# Print the end time
echo "SLURM JOB FINISHED " `date '+%Y-%m-%d %H:%M:%S'`
```

Extraction script:

```
import sys
import hyperspy.api as hs
import numpy as np
import matplotlib.pyplot as plt

############################################################
###        1. LOADING THE DATA (HSPY)        ###
############################################################

file=sys.argv[1]
filename=sys.argv[2]
master_outputdir=sys.argv[3]


#############################
# Loading the file #
#############################

print(' ')
print(">>>"+str(filename)+" processing: Initializing.<<<")
print(' ')

s = hs.load(file)
print('1.Your file was loaded:'+str(s))


####################################################
###         2. ESTIMATING ZLP CENTRE    ###
####################################################

img_size=s.axes_manager[0].size
halfsize=int(img_size/2)
s.set_signal_type('EELS')
s_zlp_mean = s.inav[halfsize:halfsize+10,1:].estimate_zero_loss_peak_centre().mean().data[0] #index in eV
index_zlp=(s_zlp_mean-s.axes_manager[2].offset)/0.75 #index zlp in bin in energy pixels


########################################################################################
###     3. Define where to start, where to end and how many windows do you want.    ###
########################################################################################
```

```
start =-10 #start index() (real value -15eV = -20*0.75eV)
end=974 #end index() (real value = 729.0eV)
step = 1 #binning in energy)

length = len(range(start,end,step))
x=np.zeros(length)
y=np.zeros(length)
iteration_index=0

####################################################
###         4. EXTRACTION PART          ###
####################################################


######################################
# 4.1 Iterate over the energy bins #
######################################

for energy_index in range(start,end,step):
    energy_start = (energy_index*0.75) #Energy value in eV.
    print(str(energy_start)+' energy_start in eV')

    ################################################
    # 4.2 Name for the folder and the output image
    ################################################

    energy_foldername =(str(energy_start).replace('.','p').replace('-','m')+'eV') #Change point to p to save the
folders
    energy_folder= str(str(master_outputdir)+str(energy_foldername)+"/")
    e_filename = str(filename)+'.tiff'

    ##############################
    # 4.3 Optional for binning in energy (not used here as step is 1)
    ##############################

    s_ti = s.isig[int((index_zlp+energy_index)):int((index_zlp+energy_index+step))].integrate1D(axis="Energy")
    print('4.Energy='+str(s_zlp_mean+energy_index)+' eV integrated.')

    #########################
    #4.4 There is a problem with the metadata of newer data when saving tiff. Delete the problematic
information:
    #########################
    s_ti.axes_manager[0].scale=1
    s_ti.axes_manager[1].scale=1
    s_ti.axes_manager[0].units=''
    s_ti.axes_manager[1].units=''

    #####################
    # 4.5 Save the image
    #####################

    s_ti2 = s_ti.as_signal2D((0,1))
    s_ti2.change_dtype('float32')
    s_ti2.save(energy_folder+e_filename, overwrite=True)
    print('5.File saved as: '+e_filename+' in '+energy_folder)


###############################################################################################
#
```

```
    # 4.6 Get the total intensity of this image and store to plot the entire spectra in the end
###########################################################################################
#

    x[iteration_index]=float((energy_index+step/2)*0.75)
    y[iteration_index]=s_ti2.integrate1D("ScanX").integrate1D("ScanY").data[0]
    iteration_index+=1
    print('')

##################################
#     END OF THE LOOP    #
##################################


######################################################################
###         5. PLOT THE RECONSTRUCTED SPECTRA         ###
### (based on the intensity of the images in each energy bin)    ###
######################################################################

plt.plot(x,y)
plt.xlabel("Energy Loss (eV)")
plt.ylabel("Intensities (a.u.)")
plt.yscale('log')
plt.title("Total intensities from the binned images")
plt.savefig(master_outputdir+"Spectra/"+filename+"_intensities.png")

print(' ')
print(' ')
print(">>>"+str(filename)+" processing: done.<<<")
print(' ')
print(' ')

###################
###     END    ###
###################
```

Reconstructing energy-loss volumes

Running script:

```
#!/bin/bash

###NOTE###
#manually edit oldparticles.star file to mirror dirname/micrographs/*tiff for micrographs
#manually edit a mics.star so that the micrographs path is micrographs/*tiff
#manually make a dirlist.txt file
#copy extractor.sh and reconstructor.sh to this directory. Make sure boxsize and other parameters are as desired in the relion commands

#run as ./script.sh in the root directory

#created by Olivia Pfeil-Gardiner on 11.07.2022
##########




###VARIABLES###
```

```
path_to_mics=/path/to/extracted_energy_micrographs

###############


#create directories

for item in `cat dirlist.txt`
do

mkdir $item
mkdir $item/micrographs
mkdir $item/particles

ln -s ${path_to_mics}/${item}/*tiff ${item}/micrographs/

cp oldparts.star $item/
cp extractor.sh $item/
cp reconstructor.sh $item/
cp oldmics.star $item/

done

for item in `cat dirlist.txt`
do
(cd $item && sbatch extract.sh)
sleep 15
done

echo DONE WITH SUBMITTING EXTRACT - wait 300
sleep 300


for item in `cat dirlist.txt`
do
(cd $item && sbatch reconstruct_pad2.sh)
sleep 30
rm -rf ${item}/micrographs/
done

echo Waiting for the reconstructions to finish..
sleep 3000

mkdir reconstructions
mkdir reconstructions/pad2

for item in `cat dirlist.txt`
do
mv $item/rec_pad2.mrc reconstructions/pad2/${item}.mrc
rm -rf $item/particles/
done
```

Extraction script:

```
#!/bin/bash

#####CAFREFUL: This script has no dependency setting! To be run with sleep xx!!###########

#SBATCH --error=Extract.err
```

```
#SBATCH --output=Extract.out
#SBATCH --partition=partition
#SBATCH --mincpus=10
#SBATCH --cpus-per-task=1
#SBATCH --ntasks=35
#SBATCH --qos=normal
#SBATCH -J »--->_Energy_Extract_<---«
#SBATCH --open-mode=append

# Print the start time
echo "SLURM JOB SUBMITTED " `date '+%Y-%m-%d %H:%M:%S'`

module load relion_own_build #module file for modified relion version

srun -n 35 `which relion_preprocess_mpi` --i mics.star --reextract_data_star oldparts.star --part_star
particles.star --pick_star extractpick.star --part_dir particles/ --extract --extract_size 120 --float16 --bg_radius 45
--white_dust -1 --black_dust -1


# Print the end time
echo "SLURM JOB FINISHED " `date '+%Y-%m-%d %H:%M:%S'`
```

Reconstruction script:

```
#!/bin/bash

###########CAREFUL: No dependencies. Use sleep xx when submitting many jobs#######

#SBATCH --error=padReco.err
#SBATCH --output=padReco.out
#SBATCH --partition=partition
#SBATCH --mincpus=1
#SBATCH --cpus-per-task=1
#SBATCH --ntasks=1
#SBATCH --qos=normal
#SBATCH -J »--->_Spectrum_Reconstruct_<---«
#SBATCH --open-mode=append

# Print the start time
echo "SLURM JOB SUBMITTED " `date '+%Y-%m-%d %H:%M:%S'`

module load relion_own_build.   #module file for modified relion version

srun -n 1 relion_reconstruct --i particles.star --o rec_pad2.mrc --sym C4 --dont_skip_gridding --pad 2

# Print the end time
echo "SLURM JOB FINISHED " `date '+%Y-%m-%d %H:%M:%S'`
```

background subtraction of volumes:

```
#!/usr/bin/env python
# coding: utf-8

import mrcfile
import numpy as np
import hyperspy.api as hs
import matplotlib.pyplot as plt

from scipy.ndimage import gaussian_filter
```

```
get_ipython().run_line_magic('matplotlib', 'notebook')


directory= '/path/to/directory/with/reconstructions/'
energies = np.genfromtxt('/path/to/file_with_all_energy_names.txt', dtype='str')


supermrc=np.zeros((len(energies),120,120,120))
i=0

for file in energies:
    s = mrcfile.open(directory+file+'.mrc').data
    sg = gaussian_filter(s,2)
    supermrc[i] = sg
    i+=1

smrc = hs.signals.Signal1D(supermrc.T)

smrc.axes_manager[3].offset=-7.5 #Note this scale is not calibrated. Apply correction factor.
smrc.axes_manager[3].scale=0.75
smrc.axes_manager[3].name = 'Energy'
smrc.axes_manager[3].units = 'eV'
smrc.axes_manager[0].units=smrc.axes_manager[1].units=smrc.axes_manager[2].units = 'Å'
smrc.axes_manager[0].scale=smrc.axes_manager[1].scale=smrc.axes_manager[2].scale = 3.65
smrc.axes_manager[0].name = 'X'
smrc.axes_manager[1].name = 'Y'
smrc.axes_manager[2].name = 'Z'
smrc.set_signal_type('EELS')


flat4d = smrc.remove_background(signal_range=(180.,264.),fast=False) #for carbon


#  Save background-subtracted volumes

size = flat4d.axes_manager[3].size

for i in range(size):
    datafloat64 = flat4d.data[:,:,:,i]
    data = np.swapaxes(np.float32(datafloat64),0,2)


    with mrcfile.new(f'/path/to/outputdir/'+energies[i]+'.mrc',overwrite=True) as filtered_mrc:
        filtered_mrc.set_data(data)
        filtered_mrc.update_header_from_data()
        filtered_mrc.update_header_stats()
        filtered_mrc.close()


# ## Save non-subtracted gaussian volumes

for i in range(size):
    datafloat64 = smrc.data[:,:,:,i]
    data = np.swapaxes(np.float32(datafloat64),0,2)


    with mrcfile.new(f'/path/to/outputdir/'+energies[i]+'.mrc',overwrite=True) as filtered_mrc:
        filtered_mrc.set_data(data)
        filtered_mrc.update_header_from_data()
```

```
    filtered_mrc.update_header_stats()
    filtered_mrc.close()
```
